# Cross-cultural adaptation, reliability and validation of the Gillette Functional Assessment Questionnaire (FAQ) into Brazilian Portuguese in patients with cerebral palsy

**DOI:** 10.1186/s12887-023-03989-0

**Published:** 2023-04-11

**Authors:** Acácia Pinheiro Alvares Fernandes da Silva, Daniela Bassi-Dibai, Brunno Lima Moreira, Aline Dalfito Gava, Henrique Yuji Takahasi, Larissa Gustinelli Pereira Belo Salomão, Marcela Cacere, Fernanda de Freitas Thomaz, Almir Vieira Dibai-Filho

**Affiliations:** 1 Postgraduate Program in Physical Education, Universidade Federal do Maranhão, Avenida dos Portugueses, 1966, Núcleo de Esportes, 1º andar, Vila Bacanga, São Luís, MA CEP 65080805 Brasil; 2 Sarah Network of Rehabilitation Hospitals, São Luís, MA Brazil; 3 Postgraduate Program in Programs Management and Health Services, Universidade Ceuma, São Luís, MA Brazil

**Keywords:** Cerebral palsy, Gait, Questionnaire, Self-report measures

## Abstract

**Background:**

The purpose of this study was to translate, cross-culturally adapt and validate the Gillette Functional Assessment Questionnaire (FAQ) into Brazilian Portuguese.

**Methods:**

The translation and cross-cultural adaptation was carried out in accordance with international recommendations. The FAQ was applied to a sample of 102 patients diagnosed with cerebral palsy (CP). Construct validity was assessed using Spearman’s correlation coefficient (rho), and the FAQ score was correlated with the Functional Mobility Scale (FMS) and Gross Motor Function Classification Scale (GMFCS). A subsample of 50 patients was used to assess reliability using intraclass correlation coefficient (ICC), standard error of measurement (SEM) and minimum detectable difference (MDD). Ceiling and floor effects were also evaluated.

**Results:**

The Brazilian version of the FAQ showed excellent test-retest reliability by the assessment of the physiotherapist (ICC = 0.99) and respondent (ICC = 0.97), as well as excellent inter-examiner reliability (ICC = 0.94). The SEM was 0.23 (physiotherapist), 0.47 (respondent) and 0.64 (inter-examiner), while the MDD was 0.64 (physiotherapist), 1.29 (respondent) and 1.76 (inter-examiner). The classification of gross motor function showed a high correlation with the FAQ applied by the physiotherapist (rho = -0.89) and by the respondent (rho = -0.87). The FMS-5 m was highly correlated with the FAQ applied by the physiotherapist and the respondent (rho = 0.88 and rho = 0.87, respectively). The FMS-50 and FMS-500 presented very high correlation with the FAQ applied by the physiotherapist (rho = 0.91 for both) and high correlation with the FAQ applied by the respondent (rho = 0.89 and rho = 0.88, respectively). The Brazilian version of the FAQ did not present the ceiling and floor effects.

**Conclusion:**

The FAQ presented adequate psychometric properties in patients with CP, indicating that it is possible to use it as a measure of functional gait mobility in Brazil.

**Supplementary Information:**

The online version contains supplementary material available at 10.1186/s12887-023-03989-0.

## Introduction

Central nervous system dysfunction in cerebral palsy (CP) results in altered motor control, leading to deviations in the pattern and delay in gait acquisition [1]. Harvey and Gorter [2] consider the quantitative analysis of gait using spatio-temporal, kinetic, kinematic and electromyographic data, performed in a movement laboratory, as the gold standard, since it generates accurate and reliable information. Systematic review conducted by Wren et al. [3] supports the use of three-dimensional gait analysis in clinical decision-making, treatment planning, and outcome assessment of orthopedic interventions in patients with neuromuscular diseases, including CP.

However, three-dimensional gait analysis does not directly measure functional mobility and, additionally, it is important to consider the impact of environmental factors on its performance. Therefore, clinical assessment of ambulation and functional mobility should be performed using a variety of measures including instrumented gait analysis, standardized assessment of motor function, and patient-reported outcome measures (PROM), family members, caregivers, and others [4].

Given the above, questionnaires and scales emerge as an important alternative. In Brazil, there are few PROMs to assess functional gait mobility in patients with CP. To the best of our knowledge, the literature presents the Functional Mobility Scale (FMS) which, when published [5], was freely translated into 7 languages, including Portuguese, but without going through a process of adaptation and validation, which guarantees cultural and semantic equivalence with the original instrument.

Other questionnaires have already been translated and validated in Brazil, but not for exclusive use in patients with CP: Pediatric Evaluation of Disability Inventory (PEDI) [6], Pediatric Evaluation of Disability Inventory – Computer Adaptive Test (PEDI-CAT) [7], Pediatric Outcomes Data Collection Instrument (PODCI) [8]. These multidimensional instruments are focused on different aspects of the activity, with different forms of administration, and consider gait assessment and transfers within the context of mobility. In addition, the Gross Motor Function Measure (GMFM-88) is a tool used to assess the gross motor capacity of children and adolescents with PC (patient’s ability to execute a task or an action, at a given moment, in a uniform or standard environment) [9]. Also, some gait tests are reliable in patients with CP, such as comfortable gait speed and fast gait speed [10].

Another tool with interesting characteristics to be applied to patients with CP is the Gillette Functional Assessment Questionnaire (FAQ). This instrument was created and validated in English for patients with chronic gait disability, presenting good internal consistency, adequate reliability and a valid construct [11]. The FAQ has been cited more than 100 times in the specialized scientific literature, reflecting wide acceptance within the clinical research community [12], however, to date, its translation and validation for other studies has not been identified in the literature languages.

For PROMs to be used in other languages and to assess the health of a given population, their proper translation, cross-cultural adaptation and analysis of psychometric properties are recommended [13,14]. This process is necessary to verify the equivalence with the original version, the instrument’s internal structure, its correlations with other questionnaires, as well as detect cultural differences and perceptions about health among populations from different countries [15].

As previously mentioned, traditional gait assessments, such as simple direct observational analysis or through video, or even performed in movement laboratories, do not reliably measure the level of gait function and do not take into account the environment in which the individual is inserted (i.e., household, school, community), nor does it consider the individual’s or family’s perception of their own performance [4]. In view of this aspect and considering the scenario of few translated and validated instruments in Brazil, this study is justified.

In addition, when compared to instruments already validated in Brazil, the FAQ presents the following positive points: assessment of the patient’s performance when walking short and long distances; environmental factors are considered (such as the use of mobility aids, stairs, unevenness, supervision of caregivers); greater adequacy to the biopsychosocial model.

Thus, the objectives of this study were: (1) to cross-culturally adapt the FAQ to the Brazilian population with cerebral palsy; (2) to examine the reliability of the FAQ; and (3) to examine the construct validity of FAQ with the FMS and Gross Motor Function Classification System (GMFCS). Our hypothesis is that the Brazilian version of the FAQ has adequate psychometric properties and is similar to the original version in the instrument.

## Methods

### Study design and ethical aspects

This is a cross-sectional study carried out according to the recommendations suggested by the Guidelines for the Process of Cross-cultural Adaptation of Self-Report Measures [14] and COnsensus-based Standards for the selection of health Measurement INstruments (COSMIN) [13]. The authorization to carry out the adaptation of the FAQ into Brazilian Portuguese was granted via e-mail by one of the authors of the questionnaire (Dr. Tom F. Novacheck).

The study was carried out in two phases: (1) translation and cross-cultural adaptation of the questionnaire and (2) analysis of the psychometric properties of the cross-culturally adapted version of the FAQ into Brazilian Portuguese. The study was approved by the Human Research Ethics Committee of the Sarah Network of Rehabilitation Hospitals (opinion number 5,402,333). All patients or those responsible for the patients validated their participation by signing the free and informed consent form.

### Setting

The research was carried out at the Sarah Network of Rehabilitation Hospitals (São Luís, Maranhão, Northeast Brazil), in the Child Rehabilitation Center, Orthopedic Facility, Movement Laboratory, Pediatric Ward and Adult Ward sectors, from December 2021 to May 2022.

### Participants

According to COSMIN [13], the minimum sample size for validation studies is 100 participants. The inclusion criteria for the present study were: patients diagnosed with CP, of both sexes and with a minimum age of 2 years. The non-inclusion criteria for the study were: patients who had undergone an orthopedic approach (surgical, Botox application or serial plaster changes) in the last 6 months; family member or caregiver unable to read and write Brazilian Portuguese; family member or caregiver without Brazilian Portuguese as a native language.

The CP diagnoses of all patients were established by pediatrician specializing in child development at the hospital, considering the definitions and classifications established by an important guiding study [16].

### Translation and cross-cultural adaptation

The process of translation and transcultural adaptation of the FAQ into Brazilian Portuguese followed the criteria of Beaton et al. [14] and was carried out in stages, as described below:

1. Translation of the questionnaire: two independent translators, both with Brazilian Portuguese as their mother tongue and fluent in English, translated the original version of the FAQ into Brazilian Portuguese.
2. Synthesis of the translations: after discussions and revisions, the two translators, under observation of one of the researchers, synthesized the two translated versions of the questionnaire and produced a single version of the FAQ in a consensual way.
3. Back-translation: two independent translators (without technical knowledge of subjects in the health area), both with English as their mother tongue and fluent in Portuguese, carried out the translation of the Portuguese version of the FAQ into English, without any previous knowledge about the original version of the questionnaire.
4. Analysis by the expert committee: four specialists in the field of rehabilitation (3 physiotherapists and 1 orthopedic physician), together with the four translators involved in the project, reviewed all translated and back-translated versions, for corrections of possible discrepancies, reaching, thus, the pre-final version of the FAQ in a manner agreed between all committee members.
5. Test of the pre-final version: the pre-final version of the FAQ was applied to 30 individuals according to the inclusion criteria and with Brazilian Portuguese as their mother tongue. The scale levels that were not understood by more than 20% of the participants would be reformulated and tested again in a new sample of 30 participants, as suggested by Rodrigues et al. [17], until the desired level of understanding was reached, thus establishing the final version of the FAQ in Brazilian Portuguese.
6. Analysis of the psychometric properties of the final version of the FAQ: in order to verify the psychometric properties of the instrument, the final version of the FAQ was applied to 102 patients for construct validity and to verify the ceiling and floor effects. In addition, a subsample of 50 patients was assessed at two times (with a mean interval of 7 days between assessments) and by two individuals (caregiver and physiotherapist).

### Gillette Functional Assessment Questionnaire (FAQ)

The FAQ is an instrument that aims to identify the usual level of gait function (Additional File 1). It is an ordinal scale, which classifies the functional level from 1 to 10, in which the value 10 corresponds to the maximum functional level (in which the patient walks, runs and climbs on flat and uneven terrain, without the need for assistance) and level 1 indicates the lowest functional capacity, in which the patient cannot walk [11].

Gross Motor Function Classification System (GMFCS).

The GMFCS was adapted and validated for Brazil by Hiratuka et al. [18]. It is an ordinal scale that has 5 different levels: level I individuals have the lowest motor function and mobility limitations, while level V individuals have the greatest limitations and have little voluntary movement.

### Functional Mobility Scale (FMS)

The FMS is a simple instrument, administered by the professional through a semi-structured interview directed to the patient and/or family members/caregivers. It is commonly used in neuromuscular disorders, including CP. It classifies the individual’s mobility in environments such as home, school and community corresponding to 3 different specific distances 5 m, 50 and 500 m, respectively, and takes into account the variety and need or not of devices to aid locomotion [5].

The FMS has 6 levels of graduation: level 1 indicates the lowest level of mobility (uses a wheelchair), while level 6 represents the highest ability to walk (independent on all surfaces, does not need support for locomotion and or need help from another person to walk on all surfaces, including uneven terrain, sidewalks, and crowded areas). The instrument also includes two other classifications: N, which indicates no classification, for example, does not complete the 500 m distance; and C, which indicates crawling to get around at home [5,19].

### Statistical analysis

For sample characterization, quantitative data were described by means of average and standard deviation (SD), and qualitative data by number and percentage. We used the intraclass correlation coefficient (ICC_2,3_) to determine test-retest and inter-examiner reliability, with its respective 95% confidence interval, standard error of measurement (SEM) and minimum detectable difference (MDD) [20]. We consider an ICC value greater than 0.75 to be adequate [21].

To determine the validity of the construct, the Spearman correlation coefficient (rho) was used in order to verify the magnitude of the correlation between the FAQ and the GMFCS (related construct) and the FMS (similar construct). We consider the following criteria for interpreting the magnitude and direction of correlations: correlations with instruments that measure similar constructs must be ≥ 0.50; correlations with instruments that measure related but different constructs should be from 0.30 to 0.50; and correlations with instruments that measure unrelated constructs should be < 0.30 ^13^.

Ceiling and floor effects were analyzed in the study. These effects are present when 15% or more of the respondents reach the minimum or maximum values as a total questionnaire score [22].

## Results

### Translation and cross-cultural adaptation

During the process of translation and cross-cultural adaptation, the structure and visual identity of the instrument were modified in order to facilitate the visualization of the items. All changes and adaptations to the FAQ were suggested by the expert committee, as described below. The term “please” (“*por favor*”) was included in the initial paragraph as a polite way of requesting that the FAQ be filled. The term “child” (“*criança*”) was replaced by “patient” (“*paciente*”) since the questionnaire is not for the exclusive use of children. The term “curbs” was initially translated as “*meio-fio*”, however, the term was adapted to “sidewalk” (“*calçada*”), given that the term “curbs” (“*meio-fio*”) receives other names in the different regions of Brazil.

The adapted version of the FAQ was applied to 30 respondents and there was an understanding of 100%. Thus, we have reached the final version of the FAQ in the Brazilian Portuguese language.

### Sample description

Table 1 shows the clinical and sociodemographic characteristics of the patients. A total of 102 patients participated in the study. The age ranged from 3 to 56 years, with a mean of 12.66 years (SD = 7.7). Most patients were female and had spastic CP. In addition, there was a predominance of diplegics and quadriplegics, with GMFCS level II and III.

**Table 1:** Personal and clinical characteristics of the sample

Variable	Number (%)
**Age**	
< 6 years	19 (18.6%)
6 to 12 years	33 (32.4%)
12 to 17 years	32 (31.4%)
> 17 years	18 (17.6%)
**Sex**	
Female	52 (51%)
Male	50 (49%)
**Topographic classification**	
Monoplegia	3 (2.9%)
Hemiplegia	22 (21.6%)
Diplegia	32 (31.4%)
Triplegia	10 (9.8%)
Quadriplegia	35 (34.3%)
**Type**	
Spastic	90 (88.2%)
Dyskinetic	7 (6.9%)
Mixed	6 (5.9%)
**GMFCS**	
I	18 (17.6%)
II	36 (35.3%)
III	30 (29.4%)
IV	11 (10.8%)
V	7 (6.9%)
**Patient’s education**	
Unschooled	8 (7.8%)
Child education	8 (7.8%)
Incomplete basic education	59 (57.8%)
Incomplete high school	14 (13.7%)
Complete high school	9 (8.8%)
Incomplete higher education	4 (3.9%)

GMFCS: Gross Motor Function Classification System

Table 2 presents the characteristics of patients according to mobility aids, physical activity and treatment history, with most of the sample using a locomotion device and some type of orthoses (46.1% using rigid ankle-foot orthoses). Most of the sample had already undergone some type of orthopedic treatment (31.4% was surgery).

**Table 2:** Description of the sample according to mobility aid, physical activity and previous treatments

Variable	Number (%)
**Locomotion assistance**	
Yes	55 (53.9%)
No	47 (46.1%)
**Orthosis**	
Yes	57 (55.9%)
No	45 (44.1%)
**Orthosis type**	
Ankle-foot (rigid)	47 (46.1%)
Ankle-foot (flexible)	6 (5.9%)
Ankle-foot (ground reaction)	2 (2%)
Knee	1 (1%)
Others	1 (1%)
**Physical activity**	
Yes	10 (9.8%)
No	92 (90.2%)
**Orthopedic treatment history**	
Yes	62 (60.8%)
No	40 (39.2%)
**Type of orthopedic treatment history**	
Botox	8 (7.8%)
Plaster	12 (11.8%)
Surgery	32 (31.4%)
Plaster and surgery	9 (8.8%)
Botox, plaster and surgery	1(1%)

Table 3 shows the classification of the sample by the FMS. Only 14.7% of the sample presented walking in the community without limitation (score 6 in the FMS) and 7.8% preferred to crawl as a form of locomotion at home. Most patients had a score of 5 in the FMS.

**Table 3:** Description of the sample according to the Functional Mobility Scale (FMS).

Level	FMS − 5 m (home)	FMS − 50 m (school)	FMS − 500 m (community)
1	10 (9.8%)	20 (19.6%)	26 (25.5%)
2	25 (24.5%)	23 (22.5%)	9 (8.8%)
3	2 (2%)	3 (2.9%)	1 (1%)
4	8 (7.8%)	6 (5.9%)	7 (6.9%)
5	33 (32.4%)	30 (29.4%)	28 (27.5%)
6	16 (15.7%)	15 (14.7%)	15 (14.7%)
C	8 (7.8%)	0 (0%)	0 (0%)
N	0 (0%)	5 (4.9%)	16 (15.7%)

C: crawling; N: not perform

Regarding the characteristics of the respondents, as shown in Table 4, most of whom were fathers and/or mothers with high school education.

**Table 4:** Characteristics of the respondents

Variable	Number (%)
Education	
Incomplete basic education	17 (16.7%)
Incomplete high school	9 (8.8%)
Complete high school	55 (53.9%)
Incomplete higher education	12 (11.8%)
Complete higher education	8 (7.8%)
Complete postgraduate	1 (1%)
**Respondents**	
Father or mother	81 (79.4%)
Grandmother or grandfather	5 (4.9%)
Uncle or aunt	3 (2.9%)
Other legal guardian	1 (1%)
Patient himself	12 (11.8%)

### Construct validity

The correlations between the instruments are described in Table 5. We confirmed the study’s hypothesis by identifying a correlation magnitude greater than 0.50 between the instruments, regardless of whether the application of the FAQ was performed by the physiotherapist or the respondent. Correlations between FAQ and GMFCS were greater than − 0.87 and between FAQ and FMS were greater than 0.87.

**Table 5:** Correlation between the Gillette Functional Assessment Questionnaire (FAQ) and the other tools used in the study

Tool	FAQ (physiotherapist)	FAQ (respondent)
GMFCS (n = 102)	rho = -0.898, p < 0.001	rho = -0.874, p < 0.001
FMS		
5 m (n = 94)	rho = 0.881, p < 0.001	rho = 0.877, p < 0.001
50 m (n = 97)	rho = 0.910, p < 0.001	rho = 0.895, p < 0.001
500 m (n = 86)	rho = 0.911, p < 0.001	rho = 0.880, p < 0.001

GMFCS: Gross Motor Function Classification System; FMS: Functional Mobility Scale

### Reliability

The test-retest reliability of the FAQ showed adequate values when considering the assessment of the physiotherapist (ICC = 0.99) and the respondent (ICC = 0.97). Inter-examiner reliability also showed an adequate value (ICC = 0.94). Other reliability information is described in Table 6.

**Table 6:** Test-retest and inter-rater reliability of the Gillette Functional Assessment Questionnaire (FAQ).

Reliability	Value
**Test-retest (physiotherapist)**	
Mean (standard deviation)	
Test	6.60 (2.77)
Retest	6.62 (2.78)
ICC	0.993
95% CI	0.988 a 0.996
SEM	0.23
SEM (%)	3.51
MDD	0.64
MDD (%)	9.78
**Test-retest (respondent)**	
Mean (standard deviation)	
Test	6.40 (2.68)
Retest	6.56 (2.71)
ICC	0.979
95% CI	0.964 s 0.988
SEM	0.47
SEM (%)	7.2
MDD	1.29
MDD (%)	19.97
**Inter-examiner**	
Mean (standard deviation)	
Test	6.89 (2.57)
Retest	7.04 (2.62)
ICC	0.946
95% CI	0.921 a 0.963
SEM	0.64
SEM (%)	9.13
MDD	1.76
MDD (%)	25.30

ICC: Intraclass correlation coefficient; CI: Confidence interval; SEM: Standard error of measurement; MDD: minimum detectable difference

### Ceiling and floor effects

When the physiotherapist applied the FAQ, 6 (5.9%) patients reached the minimum score and 15 (14.7%) patients reached the maximum score. In the respondent’s assessment, 6 (5.9%) patients reached the minimum score and 6 (5.9%) the maximum. Thus, the FAQ did not present the ceiling and floor effects (< 15%).

## Discussion

The Brazilian version of the FAQ showed excellent test-retest and inter-examiner reliability, in addition to adequate construct validity, with good applicability and understanding. No ceiling and floor effects were observed. To the best of our knowledge, this is the first cross-cultural adaptation study of the FAQ. This instrument was created in 2000 ^11^ and the literature presents its use for several clinical conditions, such as CP [23,24], osteogenesis imperfecta [25] and arthrogryposis [26]. The original study verified the reliability and validity of the FAQ [11].

We confirmed the study’s hypothesis by identifying a magnitude of correlation of the FAQ greater than 0.50 with the other instruments of this study (GMFCS, rho ≤ -0.874; FMS, rho ≤ 0.877), regardless of whether the application of the FAQ was performed by the physiotherapist or by the respondent. In the original version [11], the authors identified a correlation magnitude of the FAQ above 0.50 with the Functional Independence Measure for Children (rho = 0.635), Transfers and Basic Mobility Scale (rho = 0.764) and Global Function and Symptoms Scale (rho = 0.686).

The FAQ and FMS measure similar mobility-related constructs. However, this construct is broad and influenced by several aspects, whether intrinsic (such as the degree of spasticity) or extrinsic (such as environmental barriers) [27]. Thus, there are nuances that differentiate the two instruments: a patient can improve or worsen the mobility and remain at the same FMS score by continuing to use the same mobility aid device, while the FAQ is more sensitive to capture differences for having higher degrees of classification (from 1 to 10) [28]. However, the two scales complement each other and there is no gold standard instrument to measure the mobility of patients with CP.

The GMFCS is a measure that assesses gross motor function and this directly relates to the mobility. In this sense, our results agree with a previous study that demonstrated that GMFCS levels can be accurately predicted from the FAQ [29]. In complement, our results were similar to a study carried out in Turkey, in which negative correlations were identified between the FAQ and GMFCS when considering the evaluation of the mother (r = -0.819) and the physiotherapist (r = -0.768) [30].

Considering some differences between the validation studies of the FAQ, our study included patients with all levels (1–10) of the instrument, while the original study evaluated patients from level 6 ^11^. The sample size of the original study was smaller than the 100 patients recommended by COSMIN for construct validity analyzes [13]. Regarding reliability, the ICC values found in the present study (0.97 and 0.99 for test-retest reliability and 0.94 for inter-examiner reliability) were slightly higher than in the original study (0.92 for the test-retest reliability and 0.81 for inter-examiner reliability) [11], but all values are above the acceptable cut-off point (ICC > 0.75) [21].

Although the Portuguese version of the FMS is available for Brazil population, it did not undergo an adaptation process, which guarantees cultural and semantic equivalence with the original instrument. Recently, the FMS was translated, adapted and validated in Brazil by Davoli et al. [31], but in patients with spina bifida. COSMIN recommends that the quality of the instruments be evaluated in the target population [13], thus making it necessary to validate the FMS for Brazilian Portuguese in cerebral palsy. On the other hand, the GMFCS was translated, adapted and validated in Brazil [18] and is widely used in specialized centers.

The study has limitations that must be considered. The sample of this study was collected in a referral rehabilitation hospital, therefore, community samples or samples assisted in clinics may have particular characteristics. The Brazilian Portuguese version of the FAQ showed adequate understanding and psychometric properties, but it should be investigated in other languages. Our study only involved patients with CP and, therefore, extrapolations to other populations should be avoided. We strongly recommend that future studies verify the psychometric properties of the FAQ to evaluate patients with other clinical conditions.

## Conclusion

The Brazilian version of the FAQ showed adequate psychometric properties in patients with CP, indicating that it can be used as a functional mobility measure for functional gait assessment.

## Electronic supplementary material

Below is the link to the electronic supplementary material.

**Additional file 1** – Gillette Functional Assessment Questionnaire (FAQ): Functional Walking Scale

## Data Availability

The data and materials in this paper are available from the corresponding author on request.
