# Supplementary material for: Cross-cultural adaptation, reliability and validation of the Gillette Functional Assessment Questionnaire (FAQ) into Brazilian Portuguese in patients with cerebral palsy
Source: BMC Pediatr. 2023 Apr 11;23:165. doi: 10.1186/s12887-023-03989-0 (PMC10088132; doi:10.1186/s12887-023-03989-0)
Supplement: Supplementary file 1 — Additional file 1 – Gillette Functional Assessment Questionnaire (FAQ): Functional Walking Scale [file 12887_2023_3989_MOESM1_ESM.pdf]

English Version of the Gillette Functional Assessment Questionnaire (FAQ): Functional Walking Scale

Please, choose **one** answer below that best describes your patient's typical walking ability (with the use of any needed assistive devices).

( )	**1**	Cannot take any steps at all.
( )	**2**	Can do some stepping on his/her own with the help of another person. Does not take full weight on feet; does not walk on a routine basis.
( )	**3**	Walks for exercise in therapy and less than typical household distances. Usually requires assistance from another person.
( )	**4**	Walks for household distances, but makes slow progress. Does not use walking at home as preferred mobility (primarily walks in therapy).
( )	**5**	Walks more than 15-50 feet but only inside at home or school (walks for household distances).
( )	**6**	Walks more than 15-50 feet outside the home, but usually uses a wheelchair or stroller for community distances or in congested areas.
( )	**7**	Walks outside the home for community distances, but only on level surfaces (cannot perform curbs, uneven terrain, or stairs without assistance of another person).
( )	**8**	Walks outside the home for community distances, is able to perform curbs and uneven terrain in addition to level surfaces, but usually requires minimal assistance or supervision for safety.
( )	**9**	Walks outside the home for community distances, easily gets around on the level ground, curbs, and uneven terrain, but has difficulty or requires minimal assistance with running, climbing, and/or stairs.
( )	**10**	Walks, runs, and climbs on level and uneven terrain without difficulty or assistance.

Gillette Functional Assessment Questionnaire: Functional Walking Scale - Versão Brasileira

Por favor, escolha uma das respostas abaixo que melhor descreve a habilidade típica de andar do(a) paciente (se for o caso, com o uso de algum dispositivo para assistência ou ajuda externa).

1	Não é capaz de dar nem um passo.
2	Pode dar alguns passos com ajuda de outra pessoa. Não sustenta o peso completo sobre os pés. Não caminha regularmente.
3	Anda como exercício durante a terapia e só consegue andar distâncias menores do que os deslocamentos típicos dentro de casa. Geralmente precisa do auxílio de outra pessoa.
4	Anda dentro de casa, mas desloca-se lentamente. Andar não é sua forma preferida de locomoção dentro de casa (anda principalmente durante a terapia).
5	Anda mais do que 5 a 15 metros, mas apenas dentro de casa ou na escola (anda uma distância domiciliar).
6	Anda mais do que 5 a 15 metros fora de casa, mas geralmente usa cadeira de rodas ou carrinho para se deslocar pela comunidade ou por áreas movimentadas.
7	Anda fora de casa pela comunidade, mas apenas em superfícies planas e regulares (não consegue subir/descer calçadas, escadas ou andar em terrenos irregulares sem assistência de outra pessoa).
8	Anda fora de casa pela comunidade. É capaz de subir/descer calçadas e andar em terrenos planos e irregulares, mas geralmente precisa de mínima assistência ou supervisão para segurança.
9	Anda fora de casa pela comunidade. Consegue facilmente caminhar em terrenos planos, irregulares e subir/descer calçadas, mas apresenta dificuldades ou precisa de mínima assistência para correr, escalar e/ou subir/descer escadas.
10	Anda, corre e escala em terrenos planos e irregulares sem dificuldade e sem necessidade de assistência.
